# Predicting Chronic Hyperplastic Candidiasis Retro-Angular Mucosa Using Machine Learning

**DOI:** 10.3390/clinpract13060120

**Published:** 2023-10-28

**Authors:** Omid Moztarzadeh, Jan Liska, Veronika Liskova, Alena Skalova, Ondrej Topolcan, Alireza Jamshidi, Lukas Hauer

**Affiliations:** 1 Department of Stomatology, University Hospital Pilsen, Faculty of Medicine in Pilsen, Charles University, Alej Svobody 80, 30460 Pilsen, Czech Republic; 2 Department of Anatomy, Faculty of Medicine in Pilsen, Charles University, 32300 Pilsen, Czech Republic; 3 Sikl’s Department of Pathology, Faculty of Medicine in Pilsen, Charles University, Ed. Beneše 13, 30599 Pilsen, Czech Republic; 4 Biopticka Laboratory, Mikulasske namesti 628, 32600 Pilsen, Czech Republic; 5 Central Laboratory of Immunoanalysis, University Hospital Pilsen, Faculty of Medicine in Pilsen, Charles University, Ed. Beneše 13, 30599 Pilsen, Czech Republic; 6 Dentistry School, Babol University of Medical Sciences, Babol 4717647745, Iran

**Keywords:** machine learning, digital health, chronic mucosal lesions, leukoplakia, candidosis/chronic hyperplastic candidosis, oral intraepithelial neoplasia, oral squamous cell carcinoma

## Abstract

Chronic hyperplastic candidiasis (CHC) presents a distinctive and relatively rare form of oral candidal infection characterized by the presence of white or white–red patches on the oral mucosa. Often mistaken for leukoplakia or erythroleukoplakia due to their appearance, these lesions display nonhomogeneous textures featuring combinations of white and red hyperplastic or nodular surfaces. Predominant locations for such lesions include the tongue, retro-angular mucosa, and buccal mucosa. This paper aims to investigate the potential influence of specific anatomical locations, retro-angular mucosa, on the development and occurrence of CHC. By examining the relationship between risk factors, we present an approach based on machine learning (ML) to predict the location of CHC occurrence. In this way, we employ Gradient Boosting Regression (GBR) to classify CHC lesion locations based on important risk factors. This estimator can serve both research and diagnostic purposes effectively. The findings underscore that the proposed ML technique can be used to predict the occurrence of CHC in retro-angular mucosa compared to other locations. The results also show a high rate of accuracy in predicting lesion locations. Performance assessment relies on Mean Squared Error (MSE), Root Mean Squared Error (RMSE), R-squared (R^2^), and Mean Absolute Error (MAE), consistently revealing favorable results that underscore the robustness and dependability of our classification method. Our research contributes valuable insights to the field, enhancing diagnostic accuracy and informing treatment strategies.

## 1. Introduction

Chronic hyperplastic candidosis (CHC) is a chronic disorder of the oral mucosa [1,2,3]. Chronic oral lesions with a higher incidence include oral potentially malignant disorders (OPMDs), ulcerations of different etiology, chronic candidiasis, and the manifestation of autoimmune bullous diseases [4,5,6]. The WHO classifies OPMD conditions as those with a proven higher statistical association with oral mucosa carcinoma development compared to healthy mucosa [7,8]. Disorders are clinically mostly white, unscratchable patches and leukoplakias. Visually, these are divided into homogenous and nonhomogeneous disorders, where the second variant usually has more epithelial dysplastic changes and a worse prognosis. Nonhomogeneity is determined by hyperplasticity and the color spectrum of white–red patches [9]. Then, it is differentiated into leukoerytroplakia and erythroplakia. Disorder presence in a group of OPMDs is dynamically based on the level of actual information about its malignant transformation risk to oral carcinoma. CHC has been classified as an OPMD since the year 2017. In the fifth edition of the WHO OPMD classification, CHC was excluded due to a lack of sufficient studies proving its premalignant potential. CHC is a rare form of oral candidiasis, which has acute and chronic pseudomembranous or acute and chronic atrophic variants [10]. CHC is also clinically a white or white–red patch on the oral mucosa, so it mimics leukoplakia or erytroleukoplakia. The lesions are mostly nonhomogeneous, tough when palpitated, and can develop into a nodular form. The speckled patch of CHC is more specific, mimicking leukoerythroplakia with hyperkeratosis and atrophy [11].

The onset of CHC presents with varying incidence across the world’s regions in terms of demography, immune system health status, and so forth to a certain extent. It has also been suggested by research that many cases may not only affect people living with HIV/AIDS but also those with weakened immunity or even older people. However, it may also be observed in healthy people. Such problems as underreporting and unreliable diagnosis make its precise worldwide prevalence unclear. The development of CHC is influenced by various issues. Increased susceptibility is manifested through immunosuppression, comprising HIV infection and immunosuppressive medication usage. Other risk factors include insufficient oral hygiene, smoking, overindulgence in alcohol, and prosthetic devices in the mouth [1,2,3]. Also, some systematic conditions, like diabetes mellitus, can cause a person to be more susceptible to CHC. It is usually present in the form of white, adherent plaques or patches on the oral mucosa, such as the buccal mucosa, tongue, and palate. They are difficult to remove and usually appear with a reddened base. Patients often complain of pain, itching, burning feeling, and changes in taste. These lesions may progress to erosion or ulceration with pain and bleeding in some instances.

The diagnosis of CHC involves a clinical assessment, laboratory tests, and histopathological examination. It begins with healthcare specialists examining lesions and evaluating their patients’ history. The isolation of Candida species has been made possible through laboratory procedures like microbiological cultures and molecular techniques. Nevertheless, diagnosing this condition usually calls for histopathologic examination of biopsied tissue specimens featuring peculiar characteristics like hyperplasia of the epithelial tissues and fungal hyphae therein. The impacts of CHC may vary depending on its intensity and duration. Mild cases can result in discomfort, changes in taste perceptions, and a few aesthetic concerns. CHC can be mild and transient, but severe and prolonged cases can develop into serious complications. Candida species may continue to present for a long time and contribute to epithelial dysplasia, which, in a few very occasional cases, end with malignancy. In addition, CHC can worsen existing systematic diseases by making the quality of life poor in cases where one has weak immunity. Management of CHC is based on getting rid of the fungus, alleviating suffering, and preventing its repetition by taking into account both local and systemic factors. For mild cases, physicians often prescribe topical antifungal drugs such as nystatin or clotrimazole. Oral suspensions and topical creams for oral mucous membranes are the types of drugs utilized as mucolytics. In cases of more severe and resistant cases, systemic antifungal treatment would be required. Prescription oral drugs, including fluconazole, itraconazole, and ketoconazole, can be administered in long-run doses. This would be determined by how the patient presents and susceptibility tests performed for antifungal agents [7,8].

For effective treatment and prevention of CHC recurrence, addressing underlying risk factors is essential. Such interventions include enhancements of oral hygiene practices, control of systemic diseases such as diabetes, dose adjustments in immunosuppressive drugs, and modifications in lifestyle habits including smoking and alcohol ingestion. To determine whether the resolution of CHC has been achieved, regular check-ups after each session of treatment. Patients should also be taught about preventive measures like keeping good oral health, avoiding drinking and smoking excessively, and handling their existing health matters correctly. Candidosis is also a chronic, hyperplastic type with great consequences for its victims. Understanding its epidemiology, risk factors, clinical presentation, investigation, complications, and therapeutic approaches is crucial for any healthcare provider providing adequate and timely management to patients. Early identification and management involving antifungal therapy and controlling underlying risks can help improve patient outcomes in individuals with Candidiasis and Aspergillosis. This knowledge gap suggests that additional research needs to be carried out in order to improve our understanding of the epidemiological features and pathogenesis of CHC so as to facilitate the designing of better preventive measures and management strategies.

The research gap in this study involves the need to investigate the specific impact of anatomical locations, particularly the retro-angular mucosa, on the occurrence and development of CHC. This study aims to address this gap by utilizing machine learning techniques, specifically GBR to predict the location of CHC lesions based on important risk factors. By focusing on the retro-angular mucosa, the research seeks to gain a deeper understanding of its association with CHC development and to create a more precise predictive model for CHC lesion locations. The research gap becomes evident when considering that the existing literature may not adequately emphasize the significance of anatomical location as a predictive factor for CHC. This study intends to fill this gap by employing machine learning to enhance diagnostic accuracy and provide insights that can inform more effective treatment strategies. The findings from this research have the potential to contribute valuable insights to the field, leading to advancements in the understanding and management of CHC.

## 2. Chronic Hyperplastic Candidosis

**Figure 1:**
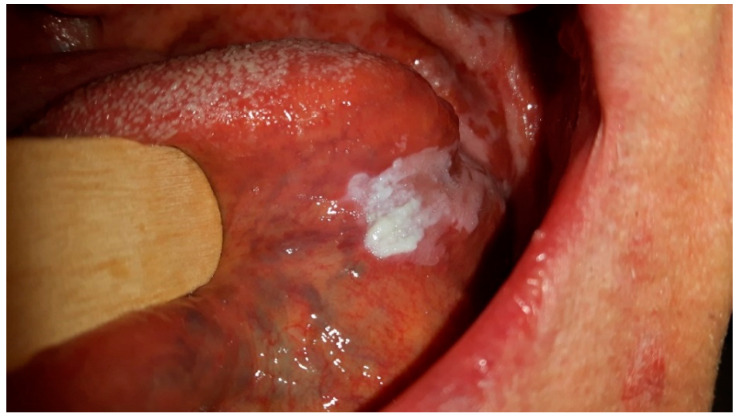
CHC predominantly located on the tongue.

**Figure 2:**
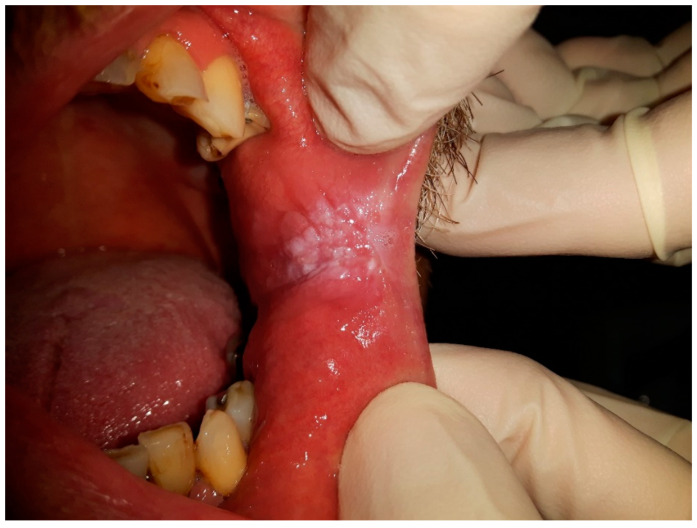
CHC presenting on retro-angular mucosa.

CHC also historically referred to as Candidal leukoplakia or Candidal leukoplakia-erythroleukoplakia, is a persistent oral mucosal condition characterized by the presence of white or cream-colored patches on the inner lining of the mouth [8,9]. These patches commonly appear on the tongue, cheek lining (buccal mucosa), and the floor of the mouth. Figure 1 shows CHC, which is located mostly on the tongue due to its surface characteristics with numerous papillae that trap debris and microorganisms, providing an environment for Candida biofilms to form. The tongue’s moist and warm environment, along with its fissures and crevices, further supports Candida overgrowth and persistence. Additionally, the reduced self-cleansing ability of the tongue’s dorsal surface and its specifics of keratinization pattern make it particularly susceptible to Candida colonization and the development of CHC lesions. While CHC can also occur in other areas of the oral mucosa, the tongue’s unique features make it a primary site for this chronic disorder. Figure 1 and Figure 2 were taken during a clinical examination at University Hospital in Pilsen for this research.

Moreover, Figure 2 demonstrates CHC occurring on the retro-angular mucosa, indicating the presence of this persistent oral mucosal condition in the retro-angular area, which is situated on the retrocommisural mucosa where the upper and lower lips meet. The retro-angular mucosa, comprising soft tissue lining, is susceptible to the overgrowth and colonization of Candida species, predominantly Candida albicans, resulting in the development of white or cream-colored patches. Similar to CHC on the tongue, the retro-angular mucosa’s warm and moist environment, along with its characteristic irregularities, creates an optimal environment for Candida biofilms to flourish. The clinical importance of CHC in this region lies in its potential to cause discomfort and irritation. Therefore, early recognition and accurate diagnosis by dental and medical professionals are crucial to initiate appropriate treatment and prevent potential complications or progression to more severe oral health concerns.

Several risk factors contribute to the development of CHC, including compromised immune system function, poor oral hygiene, wearing dentures, smoking, and certain systemic conditions like diabetes. Moreover, individuals taking immunosuppressive medications are more susceptible to CHC.

While the exact cause of CHC remains not entirely understood, it is believed to result from chronic irritation or inflammation of the oral mucosa, creating an environment favorable for Candida species to overgrow [9]. The fungus forms biofilms on the mucosal surfaces, leading to the characteristic white or cream-colored patches. Over time, these patches may become hyperplastic, indicating increased cell proliferation in the affected areas.

Diagnosing CHC involves a thorough examination of the oral cavity, and a biopsy of the affected tissue, which is mandatory to confirm the presence of Candida overgrowth and to rule out other potential conditions, such as oral squamous cell carcinoma. Treatment of CHC involves a comprehensive approach, improving oral hygiene, and employing antifungal therapy. Antifungal medications, such as nystatin or fluconazole, may be prescribed topically or systemically to eradicate the Candida infection. Regular follow-ups and monitoring are essential to prevent the condition from progressing to malignancy or causing other complications. Overall, CHC is a chronic oral mucosal disorder characterized by persistent white or cream-colored patches resulting from an overgrowth of Candida species. Due to the frequent presence of oral epithelial dysplasia in CHC biopsies, CHC necessitates careful diagnosis and management to prevent potential oral squamous cell carcinoma development. Its association with other chronic oral lesions and autoimmune blistering diseases underscores the significance of a thorough evaluation and proper treatment of these conditions to maintain oral health and overall well-being.

Lesion chronicity, its persistence to therapeutic solutions, or even progression leads clinicians to suspect a malignant process with mandatory histological examination. Histologically is CHC frequently verified with cytological atypia of increased mitotic activity and hyperplasia of basal layer cells without pleomorphism, which can only be a reactive change. A typical patient with CHC is a male, smoker, over 30 years old. Therapy of CHC consists of surgical excision, laser therapy, local or systemic antimycotics, and management of known inducing factors.

## 3. Case Study

The retrospective study included 181 patients (93 females and 88 males) who were diagnosed with CHC through histological examination. These patients received treatment at the Periodontology and Oral Medicine Department of the Dentistry Clinic at Pilsen Faculty Hospital between 1995 and 2022. The average age at the time of contact with healthcare services was 58.9 years, ranging from 25 to 90 years. Patients were eligible for inclusion in the study after their CHC diagnosis was confirmed through standard staining methods, such as periodic acid–Schiff or Grocott staining. The study also considered anamnestic and clinical risk factors, including conditions like anxiety, arterial hypertension, bronchial asthma, diabetes mellitus, gastroesophageal reflux, oral lichen planus, and thyreopathy. The concept of polymorbidity was assessed. Smoking habits were categorized into non-smokers, those smoking up to 10 cigarettes a day, and those smoking more than 10 cigarettes daily. Additionally, the study examined the use of medications known to reduce salivation and the administration of local or systemic corticosteroids. Immunological assessments, encompassing both humoral and cellular immunity, were conducted for the majority of the patients. Saliva volume was measured using the Skach test, which included evaluations of both unstimulated and stimulated salivation (each lasting 15 min, with stimulation using paraffin pellets). The study also involved pH measurements using indicator papers with a range of 4.0 to 7.5. Candida cultivation was performed through regular swabs, with differentiation between *C. albicans* and non-*albicans* yeasts accomplished using the CHROMagar system (CHROMagar, Paris, France). In this research, the machine learning approach aims to classify the location of CHC between retro-angular (RA, n = 46) and other locations (OL, n = 135), based on various parameters collected from each patient. These parameters serve as inputs to the machine learning model, and the target variable is the location of CHC, which is either retro-angular (RA) or other locations (OL).

The inputs, or features, used for the machine learning model include:

1. Sex: this categorical variable indicates the gender of each patient, with values like “Male” and “Female”;
2. Contact Age: this numerical variable represents the age of the patient at the time of contact with the medical facility;
3. Smoker: this categorical variable captures the smoking status of the patient and is divided into three subcategories—“Smoker 0” refers to successful cessation or non-smoking, “Smoker 10” represents individuals who smoke ten cigarettes per day, and “Smoker + 10” denotes patients who smoke more than ten cigarettes per day;
4. Systemic CS (Corticosteroids): this binary variable indicates whether the patient received systematic corticosteroids treatment or not, with “1” representing treatment and “0” indicating no treatment;
5. Local CS (Corticosteroids): similar to the previous parameter, this binary variable denotes whether the patient received local corticosteroids treatment or not;
6. SFR (Salivary Flow Rate—Unstimulated): this numerical variable represents the unstimulated salivary flow rate of the patient, which is measured in milliliters per minute;
7. SFR S (Salivary Flow Rate—Stimulated): similar to the previous parameter, this numerical variable denotes the stimulated salivary flow rate of the patient;
8. pH: this numerical variable represents the pH level in the patient’s saliva;
9. pH-S: similar to the previous parameter, this numerical variable represents the pH level in the patient’s stimulated saliva;
10. *C. albicans* (Colony-forming Units): this numerical variable captures the number of colony-forming units of *C. Albicans*, which is obtained from cultivation data;
11. *C.* non-*albicans* (Colony-forming Units): similar to the previous parameter, this numerical variable represents the number of colony-forming units of non-*albicans Candida* obtained from cultivation data.

The target variable, or the location of CHC, is binary and categorical, with two possible values:

1. Retro-angular (RA): this indicates the location of CHC in the retro-angular region;
2. Other Locations (OL): this indicates the location of CHC in regions other than retro-angular.

The goal of the machine learning model is to learn patterns and relationships between these input parameters and the target location, enabling accurate classification of CHC occurrences between retro-angular and other locations.

## 4. The Machine Learning Approach

Machine learning has risen as a powerful tool with a wide range of uses in understanding and controlling intricate systems across different fields [12,13,14,15]. In the field of engineering, machine learning algorithms are employed to tackle complex problems in areas like weather prediction, optimizing energy grids, and managing traffic [16,17,18,19]. These models extensively examine historical data and patterns to predict how systems will behave, detect possible bottlenecks, and improve the allocation of resources. This holds the potential to transform decision-making procedures in various sectors [20]. In various fields of research, machine learning has proven its value in addressing complex issues and functions, such as detecting fraud, evaluating risk, and executing algorithmic trading. These areas involve intricate interactions within market dynamics, necessitating advanced and sophisticated analyses [13,21,22]. As these models continuously evolve, they hold the potential to enhance complex systems and enable more efficient and adaptive decision making across diverse sectors. In the medical domain, machine learning has ushered in a new era of personalized and precision medicine [23]. The copious amount of medical information generated daily challenges traditional approaches in extracting valuable insights from this data deluge. Machine learning algorithms excel in processing and interpreting these vast datasets, playing a crucial role in early disease diagnosis, treatment optimization, and drug discovery [12,24,25,26]. Leveraging patient data, genomic information, and clinical records, machine learning identifies patterns and risk factors associated with various illnesses, leading to improved patient outcomes and cost-effective healthcare solutions [27]. Additionally, machine learning techniques are applied to automate diagnosis in medical imaging, aiding radiologists in detecting abnormalities and expediting medical interpretation. As machine learning continues to progress, its potential to transform medical research, diagnosis, and patient care remains immense, ultimately enhancing the overall healthcare landscape [16]. In the context of signal processing in medicine, Gradient Boosting Regressor (GBR) has proven to be an important method [22,28]. Its high predictive accuracy is particularly valuable when dealing with medical data, where accurate predictions can have significant implications for patient care and outcomes. Medical data can be noisy and contain missing values or outliers, and GBR’s robustness to such data makes it a suitable choice for medical applications [29].

GBR is a powerful supervised machine learning algorithm that falls under the category of ensemble methods. Ensemble methods combine the predictions of multiple individual models to make more accurate and robust predictions. The core idea behind GBR is to build an ensemble of weak learners, typically decision trees, in a sequential manner. These weak learners, often referred to as “decision stumps”, are simple and low-performing models that can only learn simple patterns in the data [28].

The training process of GBR is sequential [29]. It starts by training the first weak learner on the original data. Subsequent weak learners are then trained to correct the errors made by the previous learners. During the training process, the algorithm calculates the gradients (derivatives) of the loss function concerning the predictions made by the current ensemble. These gradients represent the direction and magnitude of the errors made by the current model. The goal of GBR is to fit the negative gradient of the loss function, hence the name “gradient boosting”, in order to reduce the overall prediction errors. Each new weak learner is trained to minimize the errors made by the current ensemble. After training each new weak learner, they are added to the ensemble with a certain weight, which depends on how much it improves the overall performance of the ensemble. The training process of GBR is an iterative one, and new weak learners are added for a specified number of iterations or until a stopping criterion is met. To make a prediction using the GBR, all the weak learner’s predictions are combined, each weighted according to its importance in the ensemble [30].

Moreover, medical data often involves complex relationships and non-linear patterns between variables. The ensemble nature of GBR allows it to capture these complex patterns effectively, enabling accurate predictions and modeling of various medical signals and parameters. The algorithm’s ability to provide insights into feature importance is beneficial in medical research, as it helps identify critical signals or patient attributes that play a crucial role in certain medical conditions or treatments. In the field of medicine, GBR finds applications in medical diagnosis and prognosis. It can predict patient outcomes, risk factors, or treatment responses based on different signals or patient data. Additionally, it supports personalized medicine by analyzing individual patient data and predicting the most effective interventions for personalized treatment plans.

GBR’s ability to handle complex and noisy medical data, its high predictive accuracy, and its feature importance analysis make it a valuable tool in signal processing and predictive modeling for various medical applications, contributing to advancements in medical research and patient care. However, it is crucial to address privacy and ethical considerations when applying machine learning algorithms to sensitive medical data. Technical parameters of the methods and materials are as follows:

A. Data Split

We have divided our dataset into a training set and a testing set. We have reported the number of samples in each set. For example, with 180 samples:

- Training Set: 70% of the data (126 samples).
- Testing Set: 30% of the data (54 samples).

B. Hyperparameters Used in GBR

We have provided the specific hyperparameters used when initializing the GBR model. Common hyperparameters and their values include:

- n_estimators: The number of boosting stages (trees) in the ensemble. For example, we have set n_estimators = 100.
- max_depth: The maximum depth of the individual trees. For example, we have defined max_depth = 5.
- learning_rate: The rate at which the model adapts to the data. For example, our choice has been learning_rate = 0.1.
- max_features: The maximum number of features considered for each split. For instance, we have used max_features = 3.
- min_samples_split: The minimum number of samples required to split a node. We have set it to min_samples_split = 2.
- min_samples_leaf: The minimum number of samples required to be at a leaf node. We have specified min_samples_leaf = 1.

Here is an example of how we have reported this information:

C. Data Division

Total samples: 180.

- Training Set: 70% (126 samples).
- Testing Set: 30% (54 samples).

D. Cross-Validation

A 5-fold cross-validation.

E. Hyperparameters Used in GBR

- n_estimators: 100.
- max_depth: 5.
- learning_rate: 0.1.
- max_features: 3.
- min_samples_split: 2.
- min_samples_leaf: 1.

These details give a clear understanding of how we have used the data, how it was divided, and the specific hyperparameters we have employed for our GBR model. This information is essential for replicating the analysis and assessing the model’s performance.

## 5. Results

The results of our study demonstrate that the GBR exhibits a strong performance in accurately classifying the location of CHC between retro-angular (RA) and other locations (OL). The GBR model’s ability to discern between these two conditions based on the provided inputs and target variables showcases its effectiveness as a classification tool.

**Figure 3:**
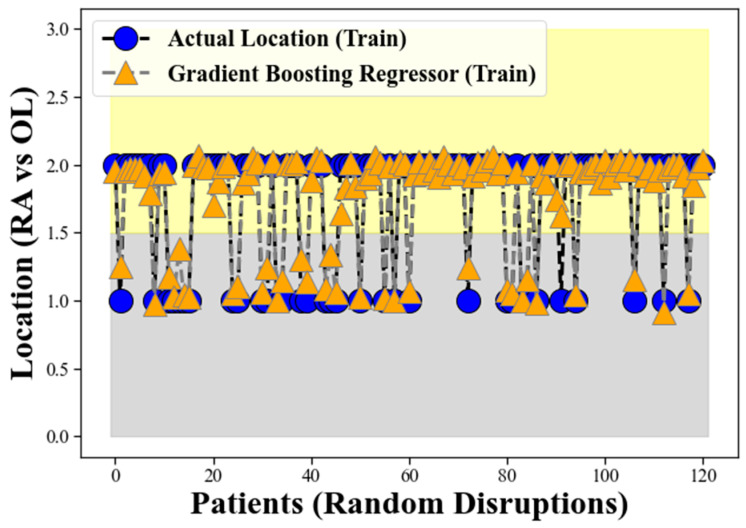
The performance of our proposed method for the classification of CHC locations.

Figure 3 illustrates the performance of the GBR model during the training process, using the input data and corresponding target labels. The orange triangular points, intersected by the grey dotted line, represent the GBR model’s response during the training phase. Conversely, the blue circles, intersected by the solid line, depict the actual location of the CHC. As evident from the figure, the GBR model’s predicted locations align remarkably well with the actual occurrences of CHC, which validates the robustness of our proposed method. The clear separation between these two bands indicates the GBR model’s proficiency in distinguishing CHC locations based on the provided input features. This spatial separation provides a solid foundation for the accurate classification of CHC instances between RA and OL, contributing to the model’s overall suitability for addressing this specific classification problem.

The performance depicted in Figure 3 reaffirms the success of our proposed method for the classification of CHC locations. The GBR’s ability to effectively handle complex and non-linear relationships in the data contributes to its high accuracy in discriminating between RA and OL. The results suggest that the GBR model has the potential to serve as a valuable tool for medical practitioners in distinguishing between these two conditions, aiding in more precise diagnoses and appropriate treatment planning for patients with CHC.

It is important to note that while our results indicate a strong performance of the GBR model, further validation on a larger and more diverse dataset would be beneficial to generalize its effectiveness across different populations. Additionally, the proposed method’s performance could be compared with other state-of-the-art machine learning classifiers to gain a comprehensive understanding of its superiority in this classification task. Nonetheless, our findings establish a promising foundation for the application of GBR in medical signal processing, specifically in the context of CHC classification, which could potentially lead to improved patient outcomes and enhanced clinical decision making in the field of oral medicine.

**Figure 4:**
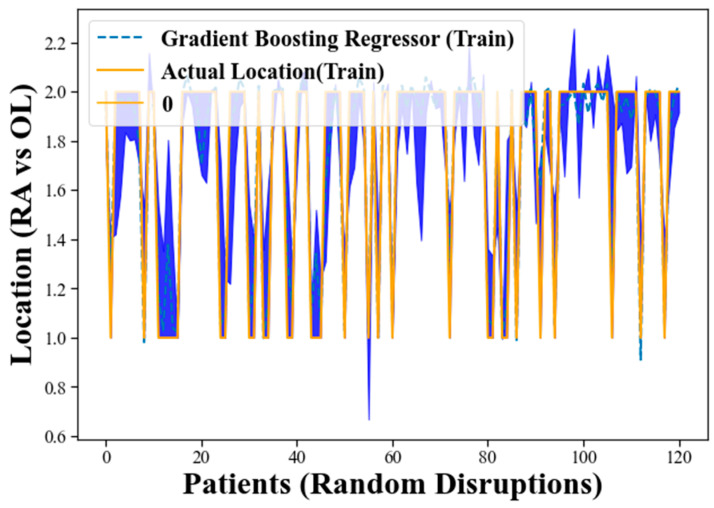
The training process for GBR using a different format.

Figure 4 presents an alternative representation of the training process for the GBR using a different format. In this visualization, the blue shadow or shaded region indicates the error between the actual measurements and the accuracy of the GBR model. The purpose of this representation is to provide insights into the model’s performance and how well it approximates the actual measurements during the training phase.

As the GBR model goes through the training process, it makes predictions for the target variable based on the provided input data. These predictions are then compared to the actual measurements of the target variable. The discrepancy or error between the predicted values and the actual measurements is depicted by the blue shadow in Figure 4.

When the GBR model’s predictions closely match the actual measurements, the blue shadow will be narrow, indicating low error. This suggests that the model is accurately approximating the target variable and performing well on the training data. On the other hand, if the predictions deviate significantly from the actual measurements, the blue shadow will be wider, indicating higher error. This implies that the model is not accurately capturing the patterns in the training data and may be overfitting or underfitting the data. In the context of Figure 4, the blue shadow’s narrow width generally indicates that the GBR model’s performance is promising. It suggests that the model is effectively learning the underlying patterns in the data and making accurate predictions for the target variable during the training process.

**Figure 5:**
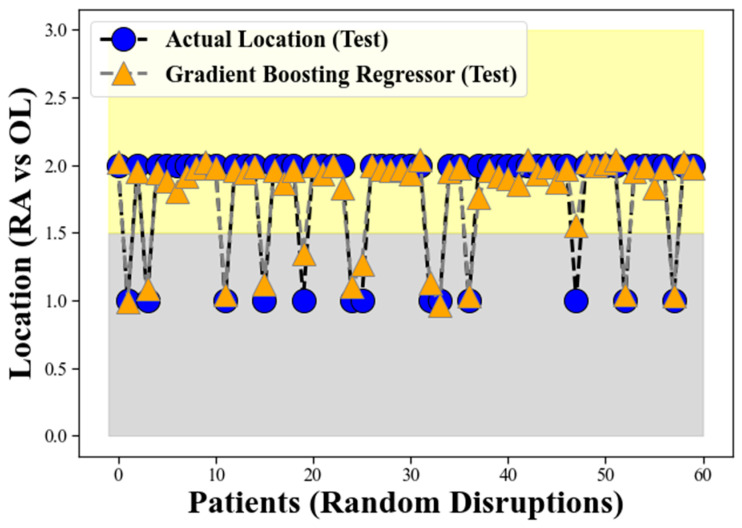
The testing process for GBR.

The grey band observed in the lower background of Figure 5 represents the locations corresponding to Retro-angular (RA), while the yellow band in the upper side signifies the occurrences of CHC in the other locations (OL) for the GBR model on the test data, similar to the representation in Figure 3. The figure illustrates the GBR model’s performance using the test data, where the orange triangular points, intersected by the grey dotted line, represent the model’s predictions on the test dataset. Conversely, the blue circles, intersected by the solid line, depict the actual location of the CHC as measured in the test data. The remarkable alignment between the GBR model’s predicted locations and the actual occurrences of CHC in the test dataset validates the robustness and effectiveness of our proposed method on unseen data.

The grey band observed in the lower background of Figure 5 continues to represent the locations corresponding to retro-angular (RA), while the yellow band on the upper side still signifies the occurrences of CHC in the other locations (OL). The clear separation between these two bands in the test data reaffirms the GBR model’s proficiency in distinguishing CHC locations based on the provided input features, even on previously unseen samples. This spatial separation demonstrates the GBR model’s ability to generalize well to new and unseen data, further supporting its suitability for accurately classifying CHC instances between RA and OL. The findings from Figure 5 provide compelling evidence of the GBR model’s strong generalization performance and its potential to be a reliable tool in medical signal processing, particularly in the context of CHC classification in oral medicine.

**Figure 6:**
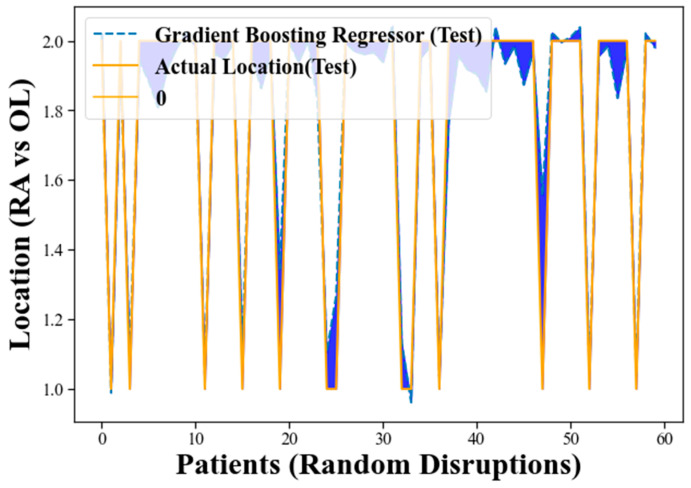
The testing process for GBR using a different format.

Figure 6 focuses on the evaluation of the GBR model using unseen test data. The blue shadow in this visualization represents the error between the GBR’s predicted values and the actual measurements of the target variable on the test dataset, as in Figure 4. A narrow blue shadow indicates that the GBR model’s predictions closely match the actual measurements, indicating low error and strong generalization to unseen data. This demonstrates the model’s ability to perform suitably on new and previously unseen data, supporting its potential for real-world applications. The promising performance displayed in Figure 5 reinforces the reliability and effectiveness of the GBR model for the classification task at hand. However, further validation and comparison with other state-of-the-art methods are essential to ascertain the model’s superiority and robustness.

In our study, we compared the performance of the GBR with three other machine learning regressors, namely Linear Regressor, Random Forest Regressor, and Decision Tree Regressor. The evaluation was conducted using various metrics to assess the accuracy and predictive power of each model. The evaluation methods employed in this comparison included Mean Squared Error (MSE), Root Mean Squared Error (RMSE), Mean Absolute Error (MAE), and R-squared (R^2^) coefficient. Mean Squared Error (MSE): MSE is a commonly used metric to measure the average squared difference between the predicted values and the actual values. A lower MSE indicates better performance, as it signifies a smaller overall error in the predictions. Root Mean Squared Error (RMSE): RMSE is the square root of the MSE and provides a measure of the average absolute error between the predicted and actual values. Similar to MSE, a lower RMSE value indicates more accurate predictions. MAE calculates the average absolute difference between the predicted and actual values. Like MSE and RMSE, a smaller MAE implies better predictive accuracy. R^2^ is a statistical measure that represents the proportion of variance in the target variable that is predictable from the independent variables. It indicates how well the model fits the data. A higher R^2^ value signifies a better fit and indicates that the model explains a larger portion of the variance in the target variable.

By comparing the results obtained from each evaluation method for the different regressors, we can determine which model performs best in accurately predicting the location of CHC between retro-angular (RA) and other locations (OL). The model with the lowest MSE, RMSE, and MAE and the highest R^2^ coefficient would be considered the most effective in this classification task. The comparison provides valuable insights into the suitability and performance of each machine learning regressor for this specific medical signal processing problem.

**Figure 7:**
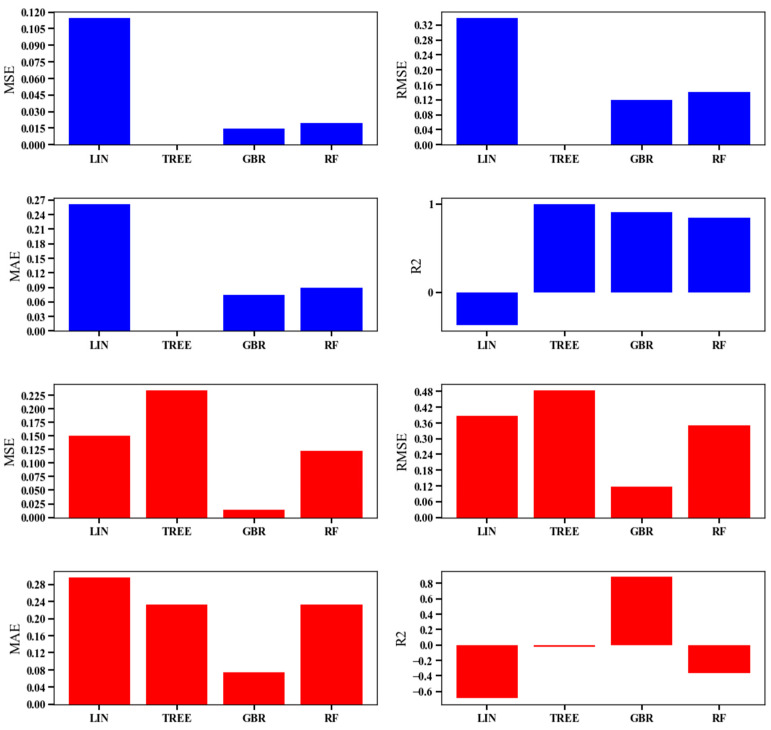
The satisfactory performance of the GBR in comparison to the other evaluated machine learning methods.

In our study, we evaluated the performance of several machine learning regressors, including GBR, Linear Regressor, Random Forest Regressor, and Decision Tree Regressor, using various evaluation metrics such as MSE, RMSE, MAE, and R^2^. Figure 7 presents the comparison results, and it indicates that the performance of GBR was deemed acceptable and promising among the evaluated methods. The GBR model demonstrated favorable results in terms of the evaluation metrics compared to the other regressors. Specifically, it achieved relatively low values for MSE, RMSE, and MAE, suggesting that the GBR model’s predictions were close to the actual values, with smaller errors compared to the other models. This indicates that the GBR model performed well in accurately predicting the location of CHC between retro-angular (RA) and other locations (OL).

Furthermore, the R^2^ coefficient for the GBR model was relatively high, implying that the model explained a significant proportion of the variance in the target variable, i.e., the CHC locations. This indicates that the GBR model’s predictions were well-fitted to the actual data, and it had a good ability to capture the underlying patterns and relationships in the dataset. The results presented in Figure 7 affirm the satisfactory performance of the GBR in comparison to the other evaluated machine learning methods. The GBR model’s ability to handle complex relationships and its robustness in predicting the target variable makes it a suitable and promising choice for the task of classifying CHC locations in medical signal processing. However, it is important to note that the choice of the best model may also depend on other factors, such as interpretability and computational efficiency, which should be considered in practical applications.

The comparison of R-A cases and those of other locations is shown in Table 1, providing a comprehensive overview of the differences in various factors related to CHC between these locations. Additionally, Table 2 presents a detailed breakdown of salivation, pH of saliva, and Candida colony-forming units (CFU) specifically for R-A and other locations cases, offering a more granular insight into the key parameters assessed in the study.

According to these tables, it was observed that males were less commonly affected by CHC, with a ratio of 0.95:1. However, in the R-A location, males had a significantly higher prevalence, with a ratio of 2.29:1. Furthermore, a substantial majority of the patients were heavy smokers (78.3%). Despite having a higher salivary volume, the pH of their saliva was decreased due to nicotine consumption, complicating the management of CHC, which often depends on quitting smoking, a challenging endeavor. Additionally, R-A cases exhibited a greater burden of both *C. albicans* and *C.* non-*albicans* colony-forming units (CFU) compared to CHC in other locations. These findings shed light on the complex interplay of gender, smoking habits, and Candida colonization in CHC cases, especially in the retro-angular mucosa region, highlighting potential implications for diagnosis and treatment.

In forthcoming investigations, the fusion of digital twin technologies, the metaverse, fuzzy systems, machine learning, Internet of Things (IoT) will present a substantial potential for addressing intricate issues [31,32,33]. This collaborative framework, reminiscent of the envisioned Meta-Metaverse, aims to enrich immersive encounters and construct precise digital duplicates spanning diverse domains [34]. Just as the layered composition introduced in the Meta-Metaverse expedites digital transformation, these amalgamated approaches could hasten our comprehension of the intricacies surrounding the reappearance of CHC. Moreover, the convergence of artificial neural networks, the metaverse, and digital twin technologies, alongside pioneering analytical methodologies, bears a revolutionary potential for deciphering the complexities underlying CHC’s recurrence. By harnessing these neural networks, we can attain more sophisticated prognostic models, refined pattern recognition, and more profound insights from intricate datasets. Comparable to the multi-tiered structure in innovative methodologies propelling digital advancement, leveraging the capabilities of artificial neural networks could significantly propel our grasp of the determinants influencing the resurgence of chronic hyperplastic candidiasis, potentially guiding more precise and efficacious interventions.

## 6. Discussion

The location of CHC lesions within the oral cavity is of great clinical significance. It not only impacts the clinical presentation but also plays a crucial role in diagnosis and treatment planning. CHC lesions can occur at various sites within the oral cavity, including the buccal mucosa, tongue, palate, and retro-angular mucosa. However, the retro-angular mucosa, also known as the retrocommissural area or the commissure of the lips, is of particular interest due to its distinct characteristics and challenges in diagnosis. The retro-angular mucosa is the area where the upper and lower lips meet at the corner of the mouth. Lesions in this location can mimic other conditions such as angular cheilitis or actinic cheilitis, leading to diagnostic confusion. Furthermore, treating lesions in this area can be challenging due to its unique anatomy and the potential for interference with lip function and esthetics.

The clinical implications of these findings point to the need for developing guidelines and best practices in managing CHC, particularly when it occurs in the retro-angular mucosa. Some potential recommendations for R-A CHC include:

- Conduct a thorough risk assessment for any CHC in patients with known risk factors, such as immunosuppression, xerostomia, or tobacco and alcohol use. Consider the retro-angular mucosa as a high-risk site for lesion development specifically in heavy smokers.
- Encourage regular oral examinations, especially for high-risk individuals. Dentists should pay close attention to the retro-angular mucosa during these exams.
- Provide education to patients about the signs and symptoms of CHC and the importance of early detection. Patients should be aware of the potential for lesions in the retro-angular mucosa.
- When a lesion is detected in the retro-angular mucosa, consider a biopsy and histopathological evaluation to confirm the diagnosis. This is particularly important due to the potential for diagnostic confusion with other conditions.
- In cases where CHC lesions in the retro-angular mucosa are challenging to manage, consider a multidisciplinary approach involving oral surgeons, and dermatologists to optimize treatment outcomes. This is also mandatory in case of systemic antimycotic usage.
- Patients with CHC, especially those with lesions in the retro-angular mucosa, should undergo long-term follow-up to monitor for recurrence and potential complications.
- Smoking should be ceased. Volume of fluid intake should be increased to compensate low pH of saliva, which should be also corrected by baking soda pills or high pH oral gels.
- It is mandatory to eradicate high *C. albicans* and *C.* non-*albicans* CFU burden.

Despite the strides made in understanding CHC, there remain several limitations and challenges. The diversity of risk factors and variations in clinical presentation complicate the diagnostic process. Additionally, the lack of a definitive biomarker for CHC diagnosis poses a significant hurdle. Histopathological examination remains the gold standard, but it requires invasive procedures and expert interpretation.

Treatment modalities for CHC are also far from standardized. Antifungal agents, such as azoles and polyenes, are commonly used, but resistance can develop over time. Surgical interventions, such as excision or laser therapy, may be necessary in refractory cases but come with their own set of complications and potential relapse. Furthermore, the specific mechanisms underlying the localization of CHC in the retro-angular mucosa remain elusive. While risk factors have been identified, the precise pathophysiological pathways leading to retro-angular CHC are not fully elucidated. This knowledge gap hinders the development of targeted preventive and therapeutic strategies.

The recent clinical findings highlighting the connection between risk factors and the location of CHC lesions in the retro-angular mucosa have significant implications for clinical practice. These findings underscore the importance of early detection, accurate diagnosis, and tailored treatment approaches for CHC, particularly when it occurs in this challenging anatomical site. The development of clinical guidelines and best practices based on these findings can enhance patient care and improve outcomes for individuals at risk of CHC. Further research in this area may provide insights into the underlying mechanisms of lesion localization and pave the way for more targeted therapies in the future. Clinicians and oral healthcare providers should remain vigilant in assessing patients with risk factors and be prepared to address CHC, especially in the retro-angular mucosa, as part of comprehensive oral healthcare.

The research has several limitations and challenges. Firstly, the diversity of risk factors and variations in clinical presentation make the diagnostic process complex. There is no definitive biomarker for CHC diagnosis, which poses a significant hurdle. While histopathological examination is the gold standard, it requires invasive procedures and expert interpretation.

Treatment modalities for CHC are not standardized. Although antifungal agents are commonly used, resistance can develop over time. Surgical interventions may be necessary in refractory cases, but they come with complications and potential relapse. Moreover, the exact mechanisms underlying the localization of CHC in the retro-angular mucosa remain unclear. While risk factors have been identified, the precise pathophysiological pathways leading to retro-angular CHC are not fully understood. This knowledge gap hinders the development of targeted preventive and therapeutic strategies.

The recent clinical findings regarding the connection between risk factors and the location of CHC lesions in the retro-angular mucosa have significant implications for clinical practice. These findings emphasize the importance of early detection, accurate diagnosis, and tailored treatment for CHC, particularly when it occurs in this challenging anatomical site. Developing clinical guidelines and best practices based on these findings can enhance patient care and improve outcomes for individuals at risk of CHC. Further research in this area may provide insights into the underlying mechanisms of lesion localization and pave the way for more targeted therapies in the future. Clinicians and oral healthcare providers should remain vigilant in assessing patients with risk factors and be prepared to address CHC, especially in the retro-angular mucosa, as part of comprehensive oral healthcare.

## 7. Conclusions

In summary, this study used GBR to predict CHC localization based on influencing factors. The GBR displayed high accuracy, highlighting machine learning’s potential in medicine. Evaluation metrics support our approach’s robustness. While specific factors are not detailed, this study advances oral pathology, aiding early diagnosis and reducing misdiagnosis.

## Figures and Tables

**Table 1:** CHC R-A and other locations. Sex differentiation, nicotinism and corticosteroid usage in R-A cases.

CHC Cases	181 Male: Female Ratio 0.95:1 (N = 88 Male, 93 Female)
R-A Cases: Other Locations	RA 25.4% (n = 46), Other locations 74.6% (n = 135)
Sex in R-A Cases	Males 69.6% (n = 32), Females 30.4% (n = 14)
Nicotinism in R-A Cases	Non-smoking 8.7% (n = 4), Smoking to 10/day 13% (n = 6), Smoking + 10/day 78.3% (n = 36)
Costicosteroid Usage in R-A Cases	Systemic corticosteroids 8.7% (n = 4), Local corticosteroids 30.4% (n = 14)

**Table 2:** Salivation, pH of saliva, Candida colony-forming units (CFU) in R-A and other locations cases.

Parameter	R-A Mean	Other Locations Mean
Salivation unstimulated	4.8 mL/15 min	3.5 mL/15 min
Salivation stimulated	22.19 mL/15 min	14.62 mL/15 min
Ph unstimulated saliva	5.92	6.38
Ph stimulated saliva	7	7.1
*C. Albicans* cfu	67.5	40.4
*C.* Non-*albicans* cfu	18.4	2.8

## Data Availability

Not applicable.
